# Stentless Management of Cannabis‐Associated STEMI in Young Adults: Two Case Reports

**DOI:** 10.1155/cric/2378215

**Published:** 2026-10-07

**Authors:** Sofiene Haboubi, Alya Mrad, Mohamed Amine Soula, Elyes Lagha, Mariem Drissa, Rami Tlili, Youssef Ben Ameur

**Affiliations:** ^1^ Cardiology Department Faculty of Medicine of Tunis University of Tunis El Manar Mongi Slim Hospital La Marsa Tunisia utm.rnu.tn

**Keywords:** cannabis, care report, myocardial infarction, stentless approach, thrombus

## Abstract

**Background:**

ST‐segment elevation myocardial infarction (STEMI) in young adults is uncommon and may occur in the absence of significant underlying atherosclerotic coronary artery disease. Recent cannabis consumption has been temporally associated with acute coronary syndromes through several proposed mechanisms, including coronary thrombosis, vasospasm, endothelial dysfunction, and platelet activation. We report two cases of STEMI occurring shortly after cannabis consumption that were managed using a stentless strategy.

**Case Presentations:**

Case 1: A 36‐year‐old man with recently diagnosed Type 2 diabetes mellitus and a history of daily cannabis use presented with an inferior STEMI approximately 1 h after cannabis consumption. Coronary angiography demonstrated double culprit lesions with thrombotic occlusion of the distal right coronary artery and a large thrombus within the proximal left anterior descending artery without significant underlying angiographic atherosclerosis. A minimalist immediate mechanical intervention (MIMI) strategy consisting of manual thrombus aspiration, dual antiplatelet therapy, and therapeutic anticoagulation was performed, followed by deferred angiographic reassessment after 7 days, which demonstrated complete thrombus resolution and TIMI 3 flow without residual significant stenosis in both vessels. Case 2: A 29‐year‐old man with heavy tobacco use and regular cannabis and alcohol consumption presented with anterior STEMI complicated by acute heart failure. Coronary angiography revealed a high thrombus burden involving the distal left main coronary artery with thrombotic occlusion of the ostial left anterior descending artery. Revascularization was achieved using manual thrombus aspiration, intracoronary glycoprotein IIb/IIIa inhibitor administration, and balloon angioplasty without stent implantation, resulting in restoration of TIMI 3 flow. No significant residual angiographic coronary stenosis was identified.

**Conclusion:**

These cases illustrate the feasibility of a stentless strategy in carefully selected young patients presenting with a high coronary thrombus burden and no angiographic evidence of significant underlying coronary artery disease. Although a temporal association with cannabis consumption was observed, a causal relationship cannot be established. These observations are hypothesis‐generating, and larger prospective studies are required to better define the mechanisms underlying cannabis‐associated STEMI and the role of stentless management in this setting.

## 1. Introduction

ST‐segment elevation myocardial infarction (STEMI) in young adults is uncommon and may result from mechanisms other than premature atherosclerotic coronary artery disease, including coronary vasospasm, spontaneous coronary artery dissection, coronary embolism, and prothrombotic conditions. Recent cannabis consumption has been reported in association with acute coronary syndromes in young individuals, although a direct causal relationship cannot be established in individual cases [1].

The management of STEMI with a high intracoronary thrombus burden and minimal underlying coronary stenosis remains challenging. In selected patients, a stentless strategy involving thrombus aspiration and adjunctive pharmacological therapy, with deferred assessment of the coronary anatomy, may avoid unnecessary stent implantation. We report two young adults presenting with STEMI shortly after cannabis consumption with coronary high thrombus burden in the absence of significant residual angiographic coronary stenosis, both managed using a stentless approach. A summary timeline of the clinical presentation, management, and outcomes of both cases is provided in Table 1. Both cases were reported in accordance with the CARE (CAse REport) guidelines. The completed CARE checklist is provided in the Supporting Information.

**Table 1:** Summary timeline of clinical presentation, management, and outcomes for Case 1 and Case 2.

Time point	Case 1	Case 2
**Baseline**	36‐year‐old man; recently diagnosed Type 2 diabetes mellitus; 5‐year history of daily cannabis use	29‐year‐old man; heavy cigarette smoking; regular cannabis and alcohol consumption
**Cannabis exposure**	Significant cannabis consumption approximately 1 h before symptom onset	Chest pain began shortly after cannabis consumption
**Presentation**	> 12 h of chest pain; inferior STEMI with transient anterior ST‐segment elevation	Anterior STEMI; Killip class II acute heart failure
**Initial angiography**	Thrombotic occlusion of distal RCA and large thrombus burden in proximal LAD	High thrombus burden in distal LMCA with ostial LAD thrombotic occlusion
**Initial treatment**	Manual thrombus aspiration; DAPT; therapeutic anticoagulation; no stent	Manual thrombus aspiration; tirofiban; balloon angioplasty; no stent
**Deferred assessment**	Repeat angiography at 7 days: complete thrombus resolution and no significant residual stenosis	Deferred reassessment proposed but declined by patient
**Follow-up**	1 year: asymptomatic, no recurrent cardiovascular events	2 years: no recurrent cardiovascular events; LVEF improved from 38% to 45%

## 2. Case Presentation

### 2.1. Case 1

A 36‐year‐old man with recently diagnosed Type 2 diabetes mellitus and a self‐reported 5‐year history of daily cannabis use presented with acute chest pain that had persisted for more than 12 h. According to the patient, symptom onset occurred approximately 1 h after significant cannabis consumption. Toxicological screening was not performed during hospitalization.

On admission, his blood pressure was 120/80 mmHg, and his heart rate was 50 beats/min. Physical examination revealed no signs of heart failure. The electrocardiogram demonstrated ST‐segment elevation in the inferior leads associated with pathological Q waves and T‐wave inversion, together with transient ST‐segment elevation in the anterior leads (Figure 1).

**Figure 1:**
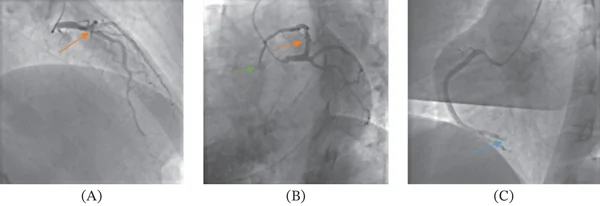
Angiographic findings in Case 1. (A) Coronary angiography showing a large thrombus burden within the proximal left anterior descending artery (arrow) with distal embolization. (B) Complete thrombotic occlusion of the distal right coronary artery (arrow). (C) Restoration of coronary flow following manual thrombus aspiration.

Transthoracic echocardiography showed moderate left ventricular systolic dysfunction with hypokinesia involving the apical, inferolateral, and septal segments.

Emergency coronary angiography demonstrated two culprit lesions: a large thrombus burden within the proximal left anterior descending (LAD) artery with distal embolization and complete thrombotic occlusion of the distal right coronary artery (RCA) (Figure 1). No significant angiographic evidence of underlying coronary atherosclerosis was identified.

Considering the patient′s young age, the high thrombus burden, and the absence of significant residual stenosis, a super‐minimalist immediate mechanical intervention (SUPER‐MIMI) strategy was adopted. Manual thrombus aspiration of the distal RCA was performed using a 6F Export Advance aspiration catheter (Medtronic, Minneapolis, MN, United States), restoring TIMI 3 flow. The patient was subsequently treated with dual antiplatelet therapy (aspirin and clopidogrel) together with therapeutic anticoagulation, with planned deferred coronary reassessment after 7 days.

The patient′s clinical course was favorable, with complete resolution of chest pain and progressive recovery of left ventricular systolic function. Repeat transthoracic echocardiography demonstrated improvement of the left ventricular ejection fraction (LVEF) to 56%. Follow‐up coronary angiography performed 7 days later demonstrated complete resolution of thrombus within both the LAD and RCA, restoration of TIMI 3 flow, and no significant residual coronary stenosis, thereby confirming that stent implantation was unnecessary (Figure 2).

**Figure 2:**
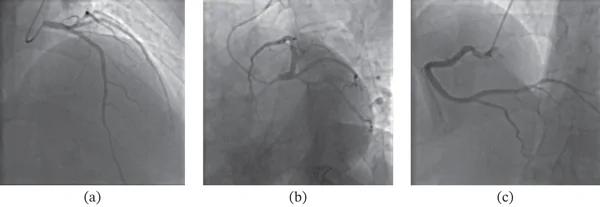
Deferred coronary angiography in Case 1 performed 7 days after the index procedure demonstrating complete thrombus resolution, restoration of TIMI Grade 3 flow, and absence of significant residual coronary stenosis in both the left anterior descending (a,b) and right coronary arteries (c).

The patient received comprehensive counseling regarding the potential cardiovascular risks associated with cannabis use and reported complete cessation of cannabis consumption following hospital discharge. At 1‐year follow‐up, he remained asymptomatic without recurrent cardiovascular events.

### 2.2. Case 2

A 29‐year‐old man with a history of heavy cigarette smoking and regular cannabis and alcohol consumption presented 3 h after the onset of severe chest pain, which had begun shortly after cannabis use according to the patient′s history. The initial presentation was complicated by Killip Class II acute heart failure. Toxicological screening was not performed during hospitalization.

Primary percutaneous coronary intervention (PCI) was undertaken through right radial artery access. Coronary angiography demonstrated a high thrombus burden involving the distal left main coronary artery (LMCA) with complete thrombotic occlusion of the ostial LAD (Figure 3). Following guidewire passage, a stentless strategy was pursued consisting of manual thrombus aspiration using an Export Advance aspiration catheter (Medtronic, Minneapolis, MN, United States), intracoronary administration of the glycoprotein IIb/IIIa inhibitor tirofiban, and adjunctive balloon angioplasty. This approach successfully restored TIMI 3 flow. Subsequent angiographic assessment demonstrated no significant residual coronary stenosis.

**Figure 3:**
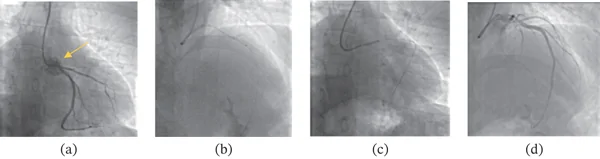
Angiographic findings in Case 2. (a) Initial coronary angiography demonstrating a large thrombus burden involving the distal left main coronary artery (arrow) with complete thrombotic occlusion of the ostial left anterior descending artery. (d) Final angiographic result following (b) manual thrombus aspiration, intracoronary glycoprotein IIb/IIIa inhibitor administration, and (c) balloon angioplasty demonstrating restoration of TIMI Grade 3 flow without significant residual stenosis.

Glycoprotein IIb/IIIa inhibitor infusion was continued for 24 h after the procedure. Transthoracic echocardiography demonstrated left ventricular dilatation with apical akinesis and moderately reduced systolic function (LVEF 38%). Guideline‐directed medical therapy for heart failure with reduced ejection fraction was initiated, including bisoprolol, ramipril, dapagliflozin, and spironolactone. The patient′s in‐hospital course was uneventful.

Following successful reperfusion and clinical stabilization, deferred coronary reassessment was proposed to evaluate the coronary anatomy after thrombus resolution and determine whether delayed stent implantation would be required. The rationale, potential benefits, and possible risks of this strategy were thoroughly discussed with the patient. Despite this counseling, the patient declined the deferred procedure because he remained asymptomatic. Shared decision‐making was therefore adopted, and optimal medical therapy was continued.

During follow‐up, the patient continued cannabis consumption despite repeated counseling regarding the potential cardiovascular risks. Nevertheless, no recurrent cardiovascular events occurred during 2 years of follow‐up, and repeat echocardiography demonstrated partial recovery of left ventricular systolic function, with improvement of the LVEF to 45%.

## 3. Discussion

Cannabis is the most widely consumed illicit psychoactive substance worldwide, and its use continues to increase following progressive legalization in several countries [2]. Parallel to this trend, increasing attention has been directed toward its potential cardiovascular effects, particularly among young adults without established coronary artery disease. Although the overall incidence remains low, observational studies and systematic reviews have reported a temporal association between recent cannabis consumption and acute cardiovascular events, including myocardial infarction, arrhythmias, stroke, and sudden cardiac death [3, 4]. Nevertheless, establishing a direct causal relationship remains challenging because many affected patients have concomitant cardiovascular risk factors or other potential triggers.

Both patients, described in the present report, developed STEMI shortly after cannabis consumption, a temporal pattern consistent with previous reports. Mittleman et al. demonstrated that the risk of myocardial infarction is highest during the first hour after cannabis use before declining rapidly thereafter [5]. Similarly, Chetty et al. reported that most published cases involve young men presenting with STEMI within a few hours of cannabis exposure [6]. Although our observations are compatible with these findings, they should be interpreted as demonstrating a temporal association rather than proving causality, particularly because the first patient had diabetes mellitus and the second had heavy tobacco use.

Several mechanisms have been proposed to explain the association between cannabis use and acute coronary syndromes. *Δ*9‐tetrahydrocannabinol, the principal psychoactive constituent of cannabis, interacts with cannabinoid receptors expressed throughout the cardiovascular system and may induce sympathetic activation, increased myocardial oxygen demand, endothelial dysfunction, oxidative stress, coronary vasoconstriction, platelet activation, and a prothrombotic state [6]. Experimental studies have also demonstrated increased expression of glycoprotein IIb/IIIa receptors and P‐selectin following cannabinoid exposure, providing a plausible biological mechanism for coronary thrombosis even in the absence of significant underlying atherosclerosis [6]. These mechanisms may act individually or synergistically, ultimately promoting thrombus formation and coronary occlusion.

However, STEMI in young adults encompasses a broad differential diagnosis that should always be considered. In addition to premature atherosclerotic disease, spontaneous coronary artery dissection, coronary vasospasm, coronary embolism, inherited or acquired thrombophilia, and inflammatory or autoimmune disorders may all present with acute coronary occlusion [1]. Although coronary angiography in our patients demonstrated a high thrombus burden without significant residual stenosis, angiography alone cannot reliably distinguish plaque rupture, plaque erosion, isolated coronary thrombosis, or other occult coronary pathology. Consequently, the absence of significant angiographic atherosclerosis should not be interpreted as definitive evidence of a nonatherosclerotic mechanism.

Intracoronary imaging using optical coherence tomography (OCT) or intravascular ultrasound (IVUS) would have provided valuable additional information regarding the underlying substrate by identifying plaque rupture, plaque erosion, spontaneous coronary artery dissection, or other structural abnormalities. Unfortunately, these imaging modalities were not available at our institution during the index procedures. Similarly, toxicological screening and investigations for inherited or acquired thrombophilia were not performed during hospitalization. Consequently, other potential prothrombotic conditions or concomitant recreational drug exposure cannot be completely excluded and should be considered when interpreting the observed temporal association with cannabis consumption.

Management of STEMI with a large intracoronary thrombus burden remains particularly challenging. Immediate stent implantation may increase the risk of distal embolization, microvascular obstruction both promoting no‐reflow, acute stent thrombosis, and unnecessary permanent coronary implantation in patients without significant residual stenosis [7]. To address these concerns, the MIMI strategy was developed, aiming to restore coronary blood flow using the least invasive mechanical approach possible while deferring definitive assessment of the culprit lesion until thrombus resolution [8].

The SUPER‐MIMI strategy extends this concept by postponing repeat coronary angiography for at least 7 days in carefully selected patients with heavy thrombus burden and minimal residual stenosis after initial reperfusion [9]. Delayed reassessment permits more accurate evaluation of the underlying coronary anatomy once thrombus has resolved and may avoid unnecessary stent implantation in patients without significant residual disease.

Evidence supporting deferred or selective stenting continues to evolve. In the INNOVATION trial, deferred stenting did not significantly reduce overall microvascular obstruction compared with immediate stenting; however, important benefits were observed in selected high‐risk patients, particularly those presenting with anterior STEMI and a large thrombus burden [10]. Similarly, the DEFER‐STEMI trial demonstrated that younger patients with substantial thrombus burden and relatively mild underlying coronary disease may derive benefit from deferred stenting through reduction of distal embolization and preservation of the coronary microcirculation [11].

Our two cases closely resemble the patient profile in whom a minimalist strategy appears most appropriate. Both patients were young, demonstrated an exceptionally high angiographic thrombus burden, and showed no significant residual coronary stenosis following restoration of TIMI grade 3 flow. In the first patient, deferred angiography confirmed complete thrombus resolution without any indication for coronary stenting. In the second patient, deferred reassessment was recommended according to the planned treatment strategy but was declined after shared decision‐making because the patient remained asymptomatic following successful reperfusion. Although these observations should not be interpreted as evidence supporting routine avoidance of stent implantation, they illustrate that a carefully selected stentless strategy may represent a reasonable therapeutic option in selected patients with predominant coronary thrombosis and no angiographic evidence of significant residual obstructive coronary disease.

Equally important, these cases emphasize that management should not focus solely on coronary revascularization. Comprehensive secondary prevention, including aggressive modification of cardiovascular risk factors and counseling regarding the potential cardiovascular consequences of cannabis consumption, remains essential. Abstinence from cannabis should be encouraged whenever a temporal relationship with an acute coronary syndrome is suspected, although further research is needed to better characterize the cardiovascular risk attributable to cannabis itself.

Several limitations should be acknowledged. First, this report describes only two patients and therefore cannot establish a causal relationship between cannabis consumption and STEMI or support recommendations regarding the routine use of a stentless strategy. Rather, these cases should be considered hypothesis‐generating.

Second, cannabis exposure was based on patient history, as toxicological screening was not performed during hospitalization. Consequently, the temporal association with cannabis consumption could not be objectively confirmed, and concomitant use of other recreational substances cannot be completely excluded.

Third, investigations for inherited or acquired thrombophilia were not performed. Although neither patient had a personal history of thromboembolic disease suggestive of an underlying hypercoagulable disorder, the possibility of an undiagnosed thrombophilic condition cannot be entirely excluded.

Fourth, intracoronary imaging with OCT or IVUS was immediately unavailable at our institution. Consequently, plaque rupture, plaque erosion, spontaneous coronary artery dissection, and other occult coronary abnormalities could not be definitively distinguished from isolated coronary thrombosis.

Finally, although both patients demonstrated favorable clinical outcomes following a stentless approach, these observations should not be interpreted as evidence of superiority over conventional primary PCI with immediate stent implantation. Larger prospective studies and dedicated registries are required to better define the pathophysiological mechanisms underlying cannabis‐associated STEMI and to determine the optimal revascularization strategy in carefully selected patients.

## 4. Conclusion

Cannabis consumption should be considered among the potential contributors to STEMI in young adults presenting with a high intracoronary thrombus burden, particularly when significant angiographic atherosclerotic disease is absent. However, the association observed in these cases does not establish causality, and alternative etiologies should always be carefully considered.

These two cases demonstrate that a carefully selected stentless strategy incorporating MIMI and intensive antithrombotic therapy may be feasible in patients with predominant coronary thrombosis and restoration of TIMI 3 flow without significant residual stenosis. Nevertheless, treatment decisions should remain individualized, and the routine avoidance of coronary stent implantation cannot be recommended based on isolated case reports.

Further prospective studies incorporating systematic toxicological evaluation, thrombophilia assessment, and intracoronary imaging are needed to better characterize cannabis‐associated STEMI and to identify patients who may safely benefit from deferred or selective stenting strategies.

## Author Contributions

**Sofiene Haboubi and Youssef Ben Ameur:** conceptualization. **Alya Mrad, Mohamed Amine Soula, and Rami Tlili:** investigation. **Mohamed Amine Soula and Alya Mrad:** data curation. **Elyes Lagha:** formal analysis. **Sofiene Haboubi and Alya Mrad:** writing – original draft. **Sofiene Haboubi, Alya Mrad, Mohamed Amine Soula, Elyes Lagha, Mariem Drissa, Rami Tlili, and Youssef Ben Ameur:** writing – review and editing. **Sofiene Haboubi and Rami Tlili:** visualization. **Youssef Ben Ameur and Sofiene Haboubi:** supervision.

## Funding

No funding was received for this manuscript.

## Disclosure

All authors contributed substantially to the work, participated in drafting or critically revising the manuscript for important intellectual content, approved the final version for publication, and agree to be accountable for all aspects of the work.

## Ethics Statement

According to the policies of our institution, formal ethical approval was not required for the publication of this retrospective case report involving two anonymized patients. All procedures performed were conducted in accordance with the ethical principles of the Declaration of Helsinki (2013 revision).

## Consent

Written informed consent for publication of the clinical details and accompanying images was obtained from both patients. Copies of the signed consent forms are available for review by the Editor upon request.

## Conflicts of Interest

The authors declare no conflicts of interest.

## Supporting information


**Supporting Information** Additional supporting information can be found online in the Supporting Information section. The CARE checklist, completed in accordance with the CARE (CAse REport) guidelines, is provided as a supporting file to document adherence to the recommended reporting items for case reports [12].

## Data Availability

The data supporting the findings of this study are available within the article. Additional anonymized clinical information is available from the corresponding author upon reasonable request, subject to institutional and ethical considerations.
