# Supplementary material for: Stentless Management of Cannabis‐Associated STEMI in Young Adults: Two Case Reports
Source: Case Rep Cardiol. 2026 Oct 7;2026:2378215. doi: 10.1155/cric/2378215 (PMC13646709; doi:10.1155/cric/2378215)
Supplement: Supplementary file 1 [file CRIC-2026-2378215-s001.docx]

**CARE Checklist of Information to Include When Writing a Case Report**

| **Topic** | **Item** | **Checklist item description** | **Reported (Page / Section)** |
| --- | --- | --- | --- |
| **Title** | 1 | The diagnosis or intervention of primary focus followed by the words “case report”. | Y — Title page (p.1): “…Two Case Reports” |
| **Key Words** | 2 | 2 to 5 key words that identify diagnoses or interventions, including “case report”. | Y — Keywords, p.2. |
| **Abstract** | 3a | Introduction: what is unique about this case and what does it add to the literature? | Y — Abstract, “Background”, p.2 |
|  | 3b | Main symptoms and/or important clinical findings. | Y — Abstract, “Case Presentations”, p.2 |
|  | 3c | The main diagnoses, therapeutic interventions, and outcomes. | Y — Abstract, “Case Presentations”, p.2 |
|  | 3d | Conclusion — what is the main “take-away” lesson(s) from this case? | Y — Abstract, “Conclusion”, p.2 |
| **Introduction** | 4 | One or two paragraphs summarizing why this case is unique (may include references). | Y — Introduction, p.2–3, refs [1]–[2] |
| **Patient Information** | 5a | De-identified patient-specific information. | Y — Case 1, p.3; Case 2, p.4 |
|  | 5b | Primary concerns and symptoms of the patient. | Y — Case 1, p.3 (chest pain >12h); Case 2, p.4 (chest pain 3h, Killip II) |
|  | 5c | Medical, family, and psychosocial history including relevant genetic information. | Partial — Case 1, p.3 (T2DM, 5y daily cannabis use); Case 2, p.4 (smoking, cannabis, alcohol). No family history reported. |
|  | 5d | Relevant past interventions with outcomes. | N/A — no prior cardiac interventions reported |
| **Clinical Findings** | 6 | Describe significant physical examination (PE) and important clinical findings. | Y — Case 1, p.3 (BP/HR/PE); Case 2, p.4 (Killip class II) |
| **Timeline** | 7 | Historical and current information from this episode of care organized as a timeline. | Y — Summary timeline table, p.11 |
| **Diagnostic Assessment** | 8a | Diagnostic testing (such as PE, laboratory testing, imaging, surveys). | Y — ECG/TTE, p.3–4; coronary angiography, p.3–4 |
|  | 8b | Diagnostic challenges (such as access to testing, financial, or cultural). | Y — Limitations paragraph, p.7 (no toxicology, thrombophilia, or OCT/IVUS testing available) |
|  | 8c | Diagnosis (including other diagnoses considered). | Y — Discussion differential-diagnosis paragraph, p.5–6 (SCAD, vasospasm, embolism, thrombophilia, inflammatory causes) |
|  | 8d | Prognosis (such as staging in oncology) where applicable. | Y — LVEF trajectory reported (Case 1, p.3; Case 2, p.4/7) as the prognostic marker used |
| **Therapeutic Intervention** | 9a | Types of therapeutic intervention (such as pharmacologic, surgical, preventive, self-care). | Y — Case 1 & 2 management, p.3–4 |
|  | 9b | Administration of therapeutic intervention (such as dosage, strength, duration). | Partial — catheter size and GP IIb/IIIa infusion duration given, p.3–4; DAPT/anticoagulant dosing not specified |
|  | 9c | Changes in therapeutic intervention (with rationale). | Y — deferred/super-MIMI rationale, p.3–4 and p.6 |
| **Follow-up and Outcomes** | 10a | Clinician and patient-assessed outcomes (if available). | Y — LVEF and symptom status, Case 1 p.3–4, Case 2 p.4/7 |
|  | 10b | Important follow-up diagnostic and other test results. | Y — 7-day angiography (Case 1), p.3; 2-year follow-up (Case 2), p.4; timeline table p.11 |
|  | 10c | Intervention adherence and tolerability (how was this assessed?). | Partial — abstinence/continued use reported by history (Case 1 abstained, Case 2 did not), p.4; assessment method not detailed |
|  | 10d | Adverse and unanticipated events. | Y — none reported, Case 1 p.3, Case 2 p.7 |
| **Discussion** | 11a | A scientific discussion of the strengths AND limitations associated with this case report. | Y — dedicated Limitations paragraphs, p.7 |
|  | 11b | Discussion of the relevant medical literature with references. | Y — Discussion, p.5–7, refs [1]–[11] |
|  | 11c | The scientific rationale for any conclusions (including assessment of possible causes). | Y — Discussion/Conclusion, p.6–7 |
|  | 11d | The primary “take-away” lessons of this case report (without references) in a one-paragraph conclusion. | Y — Discussion/Conclusion, p.6–7 |
| **Patient Perspective** | 12 | The patient should share their perspective in one to two paragraphs on the treatment(s) they received. | N — not currently included; |
| **Informed Consent** | 13 | Did the patient give informed consent? Please provide if requested. | Y — Consent section, p.8. Signed forms held by corresponding author, available on request |
